# Lidocaine Infusion: A Promising Therapeutic Approach for Chronic Pain

**DOI:** 10.4172/2155-6148.1000697

**Published:** 2017-01-11

**Authors:** Enas Kandil, Emily Melikman, Bryon Adinoff

**Affiliations:** 1 Department of Anesthesiology, University of Texas Southwestern Medical Center, Dallas, Texas, USA; 2 Department of Psychiatry, University of Texas Southwestern Medical Center, Dallas, Texas, USA

**Keywords:** Hip replacement, Knee replacement, Choice of hospital, Patient expectations, Surgery

## Abstract

Opioid abuse is a national epidemic in the United States, where it is estimated that a prescription drug overdose death occurs every 19 minutes. While opioids are highly effective in acute and subacute pain control, their use for treatment of chronic pain is controversial. Chronic opioids use is associated with tolerance, dependency, hyperalgesia. Although there are new strategies and practice guidelines to reduce opioid dependence and opioid prescription drug overdose, there has been little focus on development of opioid-sparing therapeutic approaches. Lidocaine infusion has been shown to be successful in controlling pain where other agents have failed. The opioid sparing properties of lidocaine infusion added to its analgesic and antihyperalgesic properties make lidocaine infusion a viable option for pain control in opioid dependent patients. In this review, we provide an overview of the opioid abuse epidemic, and we outline current evidence supporting the potential use of lidocaine infusion as an adjuvant therapeutic approach for management of chronic pain.

## Introduction

Chronic pain is a debilitating condition that frequently requires treatment with high doses of opioids [1]. Chronic pain affects as many as 116 million adult Americans each year with an annual estimated cost of 635 billion dollars or more in medical costs and lost wages [2]. A generalized definition of chronic pain is “Pain that extends beyond the expected period of healing” [3]. The temporal definition of chronic pain varies, but it is often described as pain that persists for more than 3 months or more than 6 months. According to the NIH, pain is one of our most pressing national public healthcare problems and as a result chronic pain was named by the NIH as the “silent epidemic” [4]. The prevalence of chronic pain is hard to assess due to the complexity of chronic pain and variance in the definition [5].

Despite advances in the specialty of pain management, chronic pain continues to be on the rise. Results from the 2012 National Health Interview Survey showed that about 25.3 million U.S. adults (11.2%) had pain almost every day for the preceding 3 months, with a staggering 40 million adults (17.6%) complained of significant or severe pain [6]. In an effort to raise awareness of the prevalence of chronic pain, the American Pain Society endorsed pain as fifth vital sign in 1999. Since this endorsement, there has been an escalating rise in the number of opioid prescriptions for chronic non-cancer pain where opioids have become the standard of care for moderate to severe pain [6–9]. This shift in practice is clearly reflected in the dramatic increase in the medical use of the four most common used opioids for pain. Since 1997, for example, morphine use has increased by 73%, hydromorphone by 96%, fentanyl by 226% and oxycodone by 403% [10]. Despite their frequent use, opioids are only partially effective for short-term pain relief and have highly variable effectiveness in the long term relief (greater than 6 months) of pain [11].

Thus far, there has been little progress on for alternative non-invasive therapeutic strategies for chronic pain patients maintained on opioids. Rightfully so, much of the effort has been focused on improving patient compliance and prescription practices. Therefore, the approach to the current opioid abuse epidemic requires not only implementation of safer practice guidelines, but also novel therapeutic approaches. In the current review, we provide an overview of the magnitude of the current epidemic of prescription opioid abuse and outline the potential of lidocaine infusion as a viable therapeutic strategy for pain control in opioid dependent patients, where lidocaine infusion has the potential to markedly reduce the dependence on opioids both in the acute and chronic settings.

## Opioid Use Disorder

In concordance with the increase of opioid prescriptions, the incidence of opioid use disorder has markedly increased. This is now one of the major health problems in the United States, with almost a daily increasing morbidity and mortality due to opioids misuse and abuse [12,13]. Recent studies clearly highlight the significant increase in opioid use, abuse, and overdose mortality due to prescription opioids [14]. There is currently an excess of 1300 deaths per year due to drug overdose involving prescription opioids. Importantly death resulting from drug overdose in general has now become the leading cause of death in the 35 to 54 age group, exceeding motor vehicle accidents [15]. This increase in opioid related complications has led the FDA to propose a risk evaluation and mitigation strategy (REMS), which applies to all long acting and immediate release opioids. In addition, this advisory panel also advocated that education for safe prescribing practices of opioids become mandatory for all prescribing physicians [16]. An important reason for the current opioid epidemic is the fact that a shift in practice in the form of leniency in opioid prescription has gradually occurred over the past two decades. This gradual shift in practice patterns has resulted in a dramatic increase in opioid sales, as well as prescription drug abuse overdoses. Despite the pressing need for formulating a comprehensive response to this problem, responding to concerns regarding opioid prescription patterns places a significant burden on providers involved in treating pain as they strive to balance the need to address the needs of their patients, and at the same time avoiding, over-prescribing, while monitoring opioids misuse and abuse [17]. Another recent advance that occurred in 2015 is that all hydrocodone-containing products officially became schedule II drugs, which immediately made it exceedingly more difficult to overprescribe, and prevented mid-level providers for administering these medications without oversight from physicians. The impact this change will have on the current opioid misuse epidemic however, remains to be seen.

### Complications associated with chronic opioid use

Chronic treatment with opioids results in a wide array of side effects including addiction, tolerance, immune modulation, as well as abnormal pain sensitivity [18]. Therefore, although opioids were initially thought to be the solution for chronic pain, opioid use has markedly exacerbated chronic pain and complicated its treatment [19]. As a result, it is now advocated that physicians adopt a far more cautious approach towards escalating opioid doses in patients suffering from chronic pain, given the large body of evidence supporting the notion that chronic opioid use of is neither safe nor effective [18].

In addition to the aforementioned complications, current evidence suggests that a more complex range of side effects that are associated with chronic opioid have been overlooked. For example, neuronal plasticity at the spinal dorsal horn level or more central in the rostroventral medulla and hippocampus results in a marked increase in pain sensitivity [20]. The effect of opioids on the neuroendocrine system has been extensively studied in animal models and in humans. Vuong et al reviewed this topic extensively, and found that although the chronic opioid changes were more relevant for opioid addiction, most of the studies highlighted acute changes in the neuroendocrine function as a result of opioid treatment. Nevertheless, the reviewed literature suggests that opioid use results in hypogonadism and weight gain by decreasing luteinizing hormone and increasing growth hormone [21]. Moreover, opioids have been known for sometime to exert an immune modulatory effect, for example morphine inhibits resistance to bacterial infection in guinea pigs [22,23]. This mechanism of this immunomodulatory effect is now quite understood, however some explanation may be provided through the expression of classic and novel opioid receptors by immune cells, which is believed to mediate the inhibitory action of opioids on proliferation of immune cells [23,24]. In addition, chronic opioid use has been shown to exert a potent immuneinhibitory effect, which is of particular concern in immune-compromised HIV infected, and the elderly patients [25]. For example, opioid use has been shown to increase the risk of pneumonia in older adults; where the odds of developing pneumonia were found to be 1.38 in elderly opioid users (95% confidence interval (CI) = 1.08–1.76) versus non-opioid users. Although the risk of these changes is highest in the first 14 days of use, there was a significant increase in risk of developing pneumonia with long-acting opioids (OR) (3.43 (95% CI = 1.44–8.21) versus non-opioid users, as well as with short-acting opioids, OR was 1.27 (95% CI = 0.98–1.64) versus non opioid users. Interestingly, in the same population the risk of pneumonia was not observed with other drugs like benzodiazepines [26].

### Chronic opioid use increases pain

In order to develop novel therapeutics for management of chronic pain, a comprehensive understanding of underlying mechanisms is critical. An important mechanism of the pathophysiology of chronic pain is the development of a maladaptive inflammatory response, which mediates pain sensation well after the initial insult is gone. For example, macrophages and lymphocytes have been shown to invade dorsal root ganglia (DRG) after acute injury in rodent models [27]. Although this is essential for the initial wound healing response, they can mediate a maladaptive response if they persist following the acute injury phase. This is partly mediated by secretion of proinflammatory cytokines, which generate spontaneous firing in sensory neurons. These spontaneous firings of sensory nerves mediate the progression of acute pain into chronic neuropathic pain [27]. Proinflammatory cytokines also play an important role in peripheral and central sensitization which causes an increase in both the duration and severity of pain [28].

**Figure 1:**
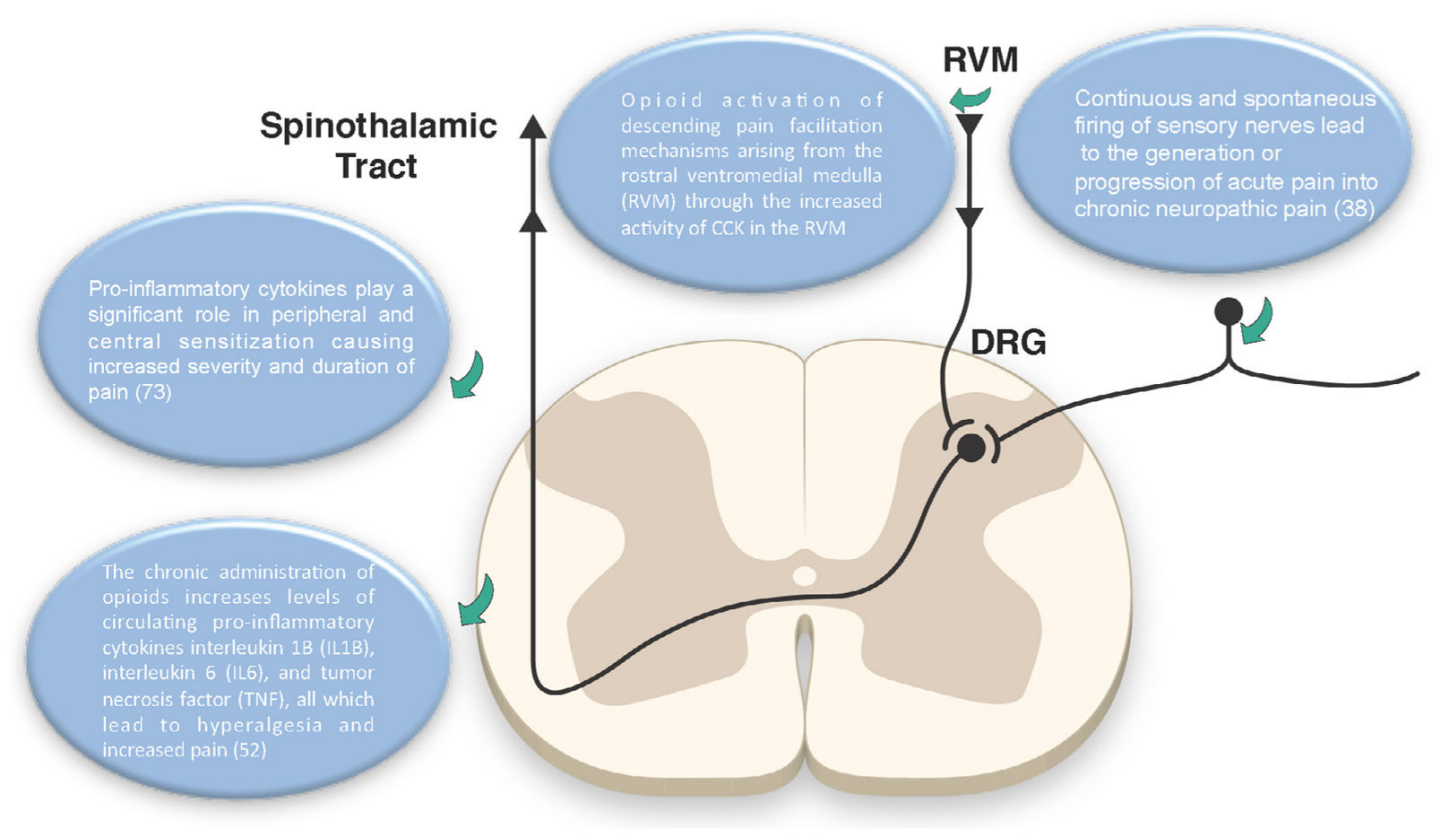
Role of opioids in development of central sensitization.

Chronic administration of opioids also results in a an increase in levels of circulating inflammatory cytokines such as interleukin 6 (IL6), interleukin 1B (IL1B), and tumor necrosis factor (TNF). The increase in these cytokines results in hyperalgesia and increased pain [29]. Although the concomitant use of anti-inflammatory drugs has been advocated to reverse this inflammatory response, their effect have proven to be suboptimal and is results in increased complications effects such renal gastrointestinal, and cardiovascular side effect, all which contribute to a significant increase in morbidity and mortality. Therefore, current evidence highlights the lack of mechanistic basis for escalating the use of opioids for chronic pain given the known effect of chronic opioid use in pain sensitization (Figure 1).

A plausible strategy to interrupt the vicious cycle of pain, inflammation and hyperesthesia is using efficacious, non-opioid medications for the treatment of chronic pain. This evidence calls for a newer pharmacotherapeutic approach that effects peripheral and central sites of action and could ultimately reverse this neuroplasticity. Such a drug could potentially relieve pain in opioid dependent patients and potentially be used as an adjuvant treatment in opioid abuse treatment programs to prevent relapse.

## Systemic Lidocaine for Treatment of Neuropathic Pain

Lidocaine infusion has been used to treat some acute and chronic pain conditions. It was first used for treatment of neuropathic pain due to burns in 1943 [30]. Since then lidocaine has only been tested in a few studies on a small number of chronic pain patients, such as patients with diabetic neuropathy and complex regional pain syndromes [31,32]. In neuropathic pain, the pathophysiology involves the modification of expression of sodium channels leading to the plasticity of responses responsible for the generation of inappropriate pain [33]. Lidocaine attenuates peripheral nociceptors sensitization and central hyperexcitability through its sodium channel blocking action [33].

**Figure 2:**
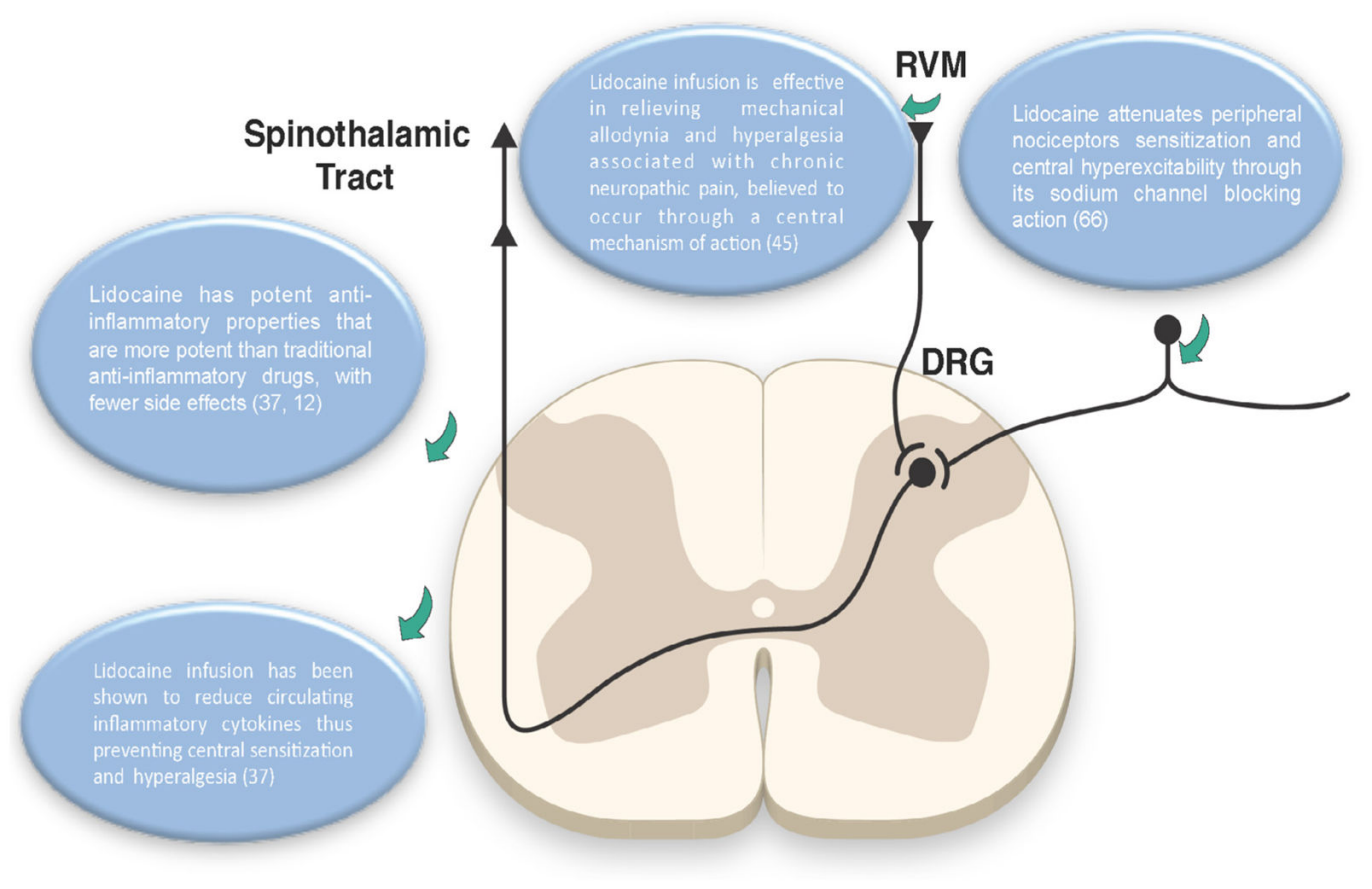
Role of lidocaine in prevention of central sensitization.

Lidocaine also has other modes of actions that explain its clinical role in treating peripheral and central pain. It has potent anti-inflammatory properties that are more potent than traditional anti-inflammatory drugs, with fewer side effects [34,35]. Through its anti-inflammatory property, lidocaine infusion has been shown to reduce circulating inflammatory cytokines [34]. The role of inflammatory cytokines is recognized in the process of secondary hyperalgesia and central sensitization [27]. Lidocaine infusion is specifically effective in relieving the mechanical allodynia and hyperalgesia associated with chronic neuropathic pain. This process is believed to occur through a central mechanism of action (Figure 2) [36].

Wallace et al. [37] evaluated the effects of I.V. lidocaine on sensory thresholds in complex regional pain syndrome (CRPS) patients. Patients received IV of lidocaine and diphenhydramine 1 week apart. The investigators measured pain scores and performed neurosensory testing. The results of this study indicated that intravenous lidocaine affects cold stimuli-related pain more significantly than mechanical pain. This demonstrates that lidocaine may primarily exert its effect on sensory processing as opposed to conduction blockade. Attal et al. [38] showed that intravenous lidocaine significantly reduced spontaneous pain and mechanical hyperalgesia. The same group also showed in a separate study [39] that lidocaine reduced neuropathic pain but did not change dynamic mechanical pain thresholds in non-neuropathic areas. Taken together, these results suggest lidocaine exerts a central modality-specific effect rather than a general pain-relieving effect. Importantly, these findings suggest that is critical to avoid reliance on visual analogue scale as a single method of assessing response to lidocaine.

In a meta-analysis, Tremont-Lukats et al. [40] noted that there is a wide variation of doses and durations of lidocaine administration for treatment of neuropathic pain, nevertheless the authors concluded that while low doses of lidocaine did not confer benefit over placebo, higher doses showed modest effect on the VAS. This meta-analysis highlights the need for standardized lidocaine administration protocols.

An important randomized, double blind, placebo-controlled clinical trial was controlled clinical trial was conducted in patients with neuropathic pain by Gottrup et al. [41]. In this study, patients were randomized to 0.24 mg/kg ketamine, to 5 mg/kg lidocaine or saline infusion, and the effect on on going or evoked pain (brush or pin-prick) was assessed. The results demonstrate that ketamine reduced both on going and evoked pain, while lidoaine only reduced pain-prick evoked pain. These results add to previous studies that highlight the complexity of the mechanism of neuropathic pain, and the need for carefully designing pain assessment techniques.

Finnerup et al. [42] assessed the role of lidocaine in spinal cord injury-associated neuropathic pain in a randomized control trial, again using a 5 mg/kg infusion protocol. The results confirmed previously observed effects of lidocain infusion on evoked pain in neuropathic pain patients, where lidocain infusion was found to decrease both evoked and spontaneous neuropathic pain. The authors concluded that the results are consistent with a central sodium-blocking effect of lidocain infusion.

Another interesting study was conducted by Viola et al43, which examine the long-term effect of lidocain infusion in patients with diabetic neuropathy. The investigators used the McGill Pain Questionnaire (MPQ) and found that lidocaine infusion markedly reduced both pain severity and quality at 14 and 28 days post infusion. This is a remarkable finding that highlights the long-lasting effect of lidocaine infusion in pain modulation that should be explored further in other indications.

In a retrospective multivariant analysis of patients that underwent lidocaine infusions, Carroll et al. [44] reported that both severity of pain and age of the patient influenced the likelihood of response to intravenous lidocaine infusion. They found that each point increase of pain (in an 11-point scale) increased the odds of responding to lidocaine by approximately 29%, while decade of life increased the odds by 36%. Not only are these results supportive of the role of lidocaine in severe pain, but also add age as an important characteristic of patients that are more likely respond to lidocaine, which can help guide future study designs.

Lidocaine infusion may be beneficial in other difficult to treat neuropathic syndromes such as fibromyalgia. A significant improvement was observed by Schafranski et al. [45] in the Fibromyalgia Impact Questionnaire FIQ scores, the Health Assessment Questionnaire, and visual analog scale (VAS) for pain. This improvement was sustained at 30 days after the last infusion. As for back pain, Park et al. [46] investigated the effects of intravenous lidocaine on neuropathic pain items of failed back surgery syndrome (FBSS) which the pain that occurs as result of abnormal impulse originated from the dorsal root ganglion and spinal cord. In this study, the authors demonstrated that 1 mg/kg, or 5 mg/kg of IV lidocaine, and placebo (attributed to small small size) improved pain in patients with neuropathic pain attributable to FBSS, however 5 mg/kg was significantly more effective. This study supports the lack of effect of low dose lidocaine infusion.

Tanen et al. [47] compared intravenous lidocaine to ketorolac for the emergency department treatment of acute radicular low back pain. Patients received either 100 mg lidocaine or 30 mg ketorolac intravenously over 2 min and changes in VAS scores was evaluated at 60 min and 1 week after treatment. In this study, the authors found that intravenous lidocaine did not improve pain associated with acute radicular low back pain. The difference between findings in this study and others previously discussed is not clear, but may be related to the protocol of infusion, or the type of pain. As outlined earlier, there is generally no consistent protocol for lidocaine infusion in the literature, which might underpin the discrepancy of observed results. The aforementioned studies are summarized in Table 1 [37–39,41–43,45–54].

### Evidence for use of systemic lidocaine for perioperative pain

Intravenous local anesthetic infusions have been used safely for pain control in the perioperative setting since the early 1950’s [55–57]. Lidocaine given intravenously in subanesthetic doses selectively blocks pain transmission in spinal cord [58], while peripherally decreasing spontaneous neuronal discharge from A delta and C fibers thus decreasing transmission of nociceptive pain [59,60]. Lidocaine has a high hepatic extraction ratio; plasma clearance is 10 ml/kg/min in patients with normal hepatic function and blood flow. Therefore, weight dosing should take into account hepatic function and hepatic blood.

Analgesia with lidocaine infusion is more effective when the intravenous lidocaine infusion is preceded by a 1–2 mg/kg bolus dose [61,62] which is likely due to achieving a faster therapeutic steady state concentration. Benefits of perioperative lidocaine infusion are range from improved VAS pain scores, opioid sparing effect and decreased hospital length of stay [62]. These benefits seem to be more important in abdominal procedures where lidocaine infusion facilitated faster return of bowel function and early hospital discharge [63,64]. These benefits suggest that lidocaine infusion is effective in relieving visceral pain, which is consistent with results seen in animal visceral pain models [65] These results are less pronounced in orthopedic procedures, cardiac surgery, and tonsillectomy cases [66]. Despite extensive research on perioperative lidocaine infusion, its benefits for non-visceral procedures, dosing, timing, and duration of infusion still need to be studied through more randomized controlled trials [66]. Table 2 outlines some of the most recent randomized controlled trials using intravenous lidocaine infusion for perioperative pain [67]. These studies are summarized in Table 2 [36,64,68–89].

### Evidence for use of systemic lidocaine for cancer pain

Despite advances in cancer treatment, there continues to be barriers for quality end of life pain management care for cancer patients. Prevalence of cancer pain varies from 33% to 64%, depending on disease stage and prognosis, and is usually rated as moderate to severe [90]. Because of the growing appreciation for the potential role of intravenous lidocaine infusion in treating refractory pain, lidocaine has been used to treat opioid refractory cancer pain in adults and children with very few and mostly self-limiting side effects [91,92]. However, the randomized controlled trials in this area are scant. In a recent phase two pilot randomized controlled cross over clinical trial, lidocaine infusion was successful in treating opioid refractory cancer pain with a mean duration of analgesia more than the half-life of lidocaine (9.34 days) ± 2.58 after a single infusion [93].

Intravenous lidocaine infusion is an appealing option in opioid refractory cancer pain as it is inexpensive, and easy to administer. In addition, lidocaine analgesia is no associated tolerance with repeated administration, does not depend on source of pain, can be repeated as needed, and allows for discontinuation of other analgesic with consequent drug related side effects [94]. However, lidocaine infusion is not mainstream treatment for opioid refractory cancer pain as phase 4 clinical trials are needed to establish guidelines for treatment in opioid refractory pain [93]. Lidocaine toxicity at small doses has been reported in terminally ill patients despite normal liver and renal function, suggesting altered pharmacodynamics [95]. These studies are summarized in Table 3 [93,96–98].

## Conclusion

In the current review, we provide a comprehensive overview of the large body of literature outlining the mechanism of action and role of lidocaine infusion in treatment of pain. Although the literature reviewed strongly supports the role of lidocaine infusion as a pain management modality, the studies reviewed vary widely in study design, patient populations, methods of pain testing, and outcomes.

Lidocaine is an amide local anesthetic with a wide range of mechanisms of action. Lidocaine, when given in a low dose intravenous infusion, successfully provides pain relief in several chronic painful conditions that have failed other treatment modalities. Lidocaine infusion is an inexpensive and relatively easily administered treatment that has been safely used with very few side effects. Lidocaine as an infusion has opioid sparing effects, blocks sodium channels, uncouples G protein, blocks NMDA receptor, reduces circulating inflammatory cytokines, and prevents secondary hyperalgesia and central sensitization.

Lidocaine infusion has been studied extensively for perioperative pain control with contradicting outcomes. These conflicting results are likely due to the limited number of patients in each study and due to the lack of standardization of study techniques. There is a paucity of studies that have assessed differences in dose, infusion protocol and adverse effects of lidocaine administration. Lidocaine infusion has been successful in treating opioid refractory pain in cancer pain patients; however randomized controlled trials are lacking. Despite its opioid sparing effect, the role of lidocaine infusion in modulating opioid dependence and addiction in patients with chronic pain is yet to be determined. Several unanswered questions need to be addressed before lidocaine infusion can be used as a mainstream treatment; including the precise dosing regimen, infusion duration and the appropriate patient selection criteria. If proven effective, lidocaine infusion can potentially be an important tool for treatment of opioid dependence.

## Figures and Tables

**Table 1:** Summary of randomized trials of lidocaine infusion for neuropathic pain.

Type of pain	Study	Condition treated	Method	Number of subjects	Intervention	Outcome	Conclusion	Adverse events
Central	Attal et al.	Neuropathic pain	Randomized double blind Crossover, 3-wk washout	16	IV Lidocaine, 5 mg.kg^−1^, 30 min	Intensity of spontaneous ongoing pain	Lidocaine > Placebo Supraspinal mechanisms of lidocaine actions are demonstrated by its effectiveness in hemispheric lesions and central pain.	Lightheadedness (44%)
Central	Kvarnstrom,	Spinal Cord Injury with Pain Below Injury Level	Randomized, double-blind three treatment Crossover	10	IV lidocaine 2.5mg.kg^−1^, 40 min Ketamine (0.4 mg/kg IV) vs. Lidocaine (2.5 mg/kg IV) vs. Placebo (NS) over 40 min	visual analogue scale (VAS). Sensory function, sensory tests and temperature thresholds.	Lidocaine = Placebo No significant difference in response between both groups in VAS spontaneous pain scores and evoked allodynia.	4/10 Drowsiness, perioral paresthesia 5 somnolence, 1 dizziness, 2 out of body sensation, change in hearing, 2 paresthesias.
Peripheral	Kvarnstrom	Long lasting, Posttraumatic, neuropathic pain	Randomized double blind, crossover	12	Ketamine (0.4 mg/kg IV) vs. Lidocaine (2.5 mg/kg) vs. placebo infused over 40 min	Visual analogue scale (VAS) Warm and cold perception as well as heat and cold pain thresholds Sensibility to touch was also tested	Lidocaine = Placebo No significant difference in VAS resting score between lidocaine and placebo; no significant difference in any evoked VAS scores	9 somnolence, 5 light-headedness,4 “out of body sensation”, 3 nausea, 2 pruritus, 2 paresthesia.
Central	Finnerup	Spinal cord injury	Crossover Double-blind Placebo controlled	24	IV Lidocaine 5 mg.kg^−1^ or placebo, 30 min	Visual Analog Scale and quantitative sensory testing.	Lidocaine > Placebo For pain at and below the level of injury irrespective of the presence or absence of evoked pain.	11 somnolence, 7 dizziness, 7 dysarthria, 7 lightheaded, 3 blurred visions.
Peripheral	Tremont-Lukats	Peripheral neuropathic pain	Parallel	32	1, 3, and 5 mg.kg.hr^−1^, 6 h		Lidocaine > Placebo	Placebo: 6/7 lidocaine (all doses): 21/23.
Peripheral	Wallace	Complex regional pain syndrome (CRPS I and II) with a prominent allodynia	Crossover 1 week washout	16	IV lidocaine and diphenhydramine separated by 1 week by increases in plasma levels of lidocaine of 1, 2, and 3 μg.ml^−1.^	Spontaneous and evoked pain scores, neurosensory testing within the painful area was measured.	Lidocaine caused a significant elevation of the hot pain thresholds in the painful area and decreased response to stroking and cool stimuli in the allodynic area. Significant decrease in pain scores to cool stimuli at all plasma levels and the spontaneous pain at the highest plasma level.	Delirium, nausea
Peripheral	Wu	Post-amputation pain: phantom limb or stump pain	Crossover, 24-h washout	32 11 cases Stump pain alone, 9 cases phantom pain alone, and 11 with both	IV Lidocaine 1 mg.kg^−1^ bolus, followed a 4 mg.kg^−1^ infusion vs. morphine 0.5 mg.kg^−1^ bolus 0.02 mg.kg^−1^ infusion vs. active placebo diphenhydramine, 10 mg bolus IV 40 mg infusion). All infusions lasted 40 min.	Phantom and stump pain ratings, sedation scores, and 0–100 visual analog scale (VAS).	Lidocaine > Placebo for stump but not phantom pain. Lidocaine = Morphine > Placebo in self-reported ratings of pain and satisfaction for stump pain.	No adverse events reported. 1/32 withdrawn because of no pain before treatment.
Peripheral	Attal	Pain-related to postherpetic neuralgia or traumatic nerve injury	Randomized controlled double-blind, Crossover	22	IV Lidocaine 5 mg.kg-1 30 min vs. placebo while 16 patients subsequently received mexiletine on an open basis titrated from 400 to 1,000 mg per day (mean 737 mg/day).	Spontaneous pain and Quantitative sensory testing. Change in mechanical dynamic allodynia and static (punctate) mechanical allodynia/hyperalgesia, but not thermal allodynia and hyperalgesia.	Five of 22 patients were pain free with lidocaine, 11 of 22 had 50% reduction of spontaneous pain, and 12 of 22 had 33% reduction of spontaneous pain.	Somnolence, lightheadedness and perioral numbness, which were present in 16 of 22 patients.
Peripheral	Lemming	PNP Whiplash disorder	Crossover	33	IV Lidocaine 5 mg.kg-1 30 min Ketamine (0.3 mg/kg infused over 30 min) vs. Lidocaine vs. morphine vs. placebo (NS)		No significant difference in response between all treatment arms; all treatment arms did illicit partial response	No reported adverse events
Peripheral	Viola	Diabetic PNP Previous responders to lidocaine	Crossover Double-blind, Placebo controlled 4 week wash out	15	5 and 7.5 mg.kg^−1^, 4 h Lidocaine (5 mg/ml IV) vs. Lidocaine (7.5 mg/ml IV) vs. placebo (NS), 5ml/kg over 4 h × 1 each four week washout.	Pain perception with McGill Pain Questionnaire (MPQ), hours of sleep, fasting blood glucose, and use of other pain-relieving medication.	Lidocaine > Placebo Both doses of lidocaine decreased MPQ resting pain scores compared to placebo; effect lasted up to 28 days post-infusion.	1 patient reported lightheadedness with 7.5 mg/ml infusion
Peripheral	Gottrup	Nerve injury pain	Randomized Placebo-controlled Crossover	20	IV infusion of ketamine (0.24 mg.kg^−1^), lidocaine (5 mg. kg^−1^), or saline for 30 min.	Effects on spontaneous and mechanical evoked pain.	Lidocaine only reduced evoked pain to repetitive pinprick stimuli. Ketamine was superior to lidocaine in reducing spontaneous pain.	Sixteen patients (84%) experienced side effects from lidocaine, compared with 11% in the placebo group.
Peripheral	Gormsen	PNP chronic neuropathic pain (peripheral nerve injury)	Randomized double-blind, placebo-controlled, three-way crossover	13	IV lidocaine 5 mg/kg vs. NS1209 (AMPA Receptor antagonist 322 mg total) vs. placebo (NS) over 4 h	Spontaneous current pain and pain evoked by brush, pinprick, cold, and heat stimulation	No difference in any treatment arms of spontaneous current pain, both NS1209 and lidocaine exhibited significant effects on resting pain compared to placebo	Drowsiness, perioral paresthesia, headache, dizziness, fatigue, discomfort, dry mouth, nausea, muscle spasm.
Peripheral	Schafranski	PNP Fibromyalgia	Open	23	IV lidocaine 2–5 mg/kg 2 h. Five sequential intravenous 2% lidocaine infusions with rising dosages (2–5 mg/kg, days 1–5).	Fibromyalgia Impact Questionnaire (FIQ), Health Assessment Questionnaire, and a visual analog scale (VAS) for pain were applied before the first lidocaine infusion, immediately after the fifth infusion, and 30 days after the fifth infusion.	A significant improvement was observed in the FIQ scores after the fifth infusion and maintained after 30 days.	No adverse events reported.
Peripheral	Park	Failed back surgery syndrome (FBSS)	Randomized controlled double-blind Crossover, 2 weeks wash out	18	Patients received each of following intravenous infusion over 1 hour at 2 weeks apart: normal saline placebo, lidocaine 1 mg/kg, and lidocaine 5 mg/kg at 60 ml/hr initially, and then titrated infusion speed while keep the heart rate <130 rates/minor 160 mmHg >systolic blood pressure >85 mmHg.	VAS and neuropathic pain questionnaire.	Lidocaine = Placebo in controlling neuropathic pain- related to FBSS.	No adverse events reported.
Peripheral	Schipper	Peripheral neuropathic pain	prospective, uncontrolled, open-label	16	IV lidocaine (5 mg.kg^−1^) within 30 min followed by long-term oral Oxcarbazepine (900–1,500 mg.day^−1^)	Daily numeric pain scores for a period 28 days.	Prematurely aborted due to ethical reason.	Lidocaine infusion well tolerated with slight paresthesias and dizziness. 6 out of 16 participants (38%) discontinued oxcarbazepine treatment due to side effects.
Peripheral	Tanen	Acute radicular back pain	Randomized controlled double-blind Crossover	21 lidocaine, 20 ketorolac	100 mg lidocaine or 30 mg ketorolac intravenously over 2 min.	A 100-mm visual analog scale (VAS) at Time 0 (baseline), and 20, 40, and 60 minutes and 1 week.	Intravenous lidocaine failed to clinically alleviate the pain associated with acute radicular low back pain.	1 in the ketorolac and 1 in the lidocaine group withdrew at the 20-min time point due to a lack of improvement in symptoms.

**Table 2:** Summary of randomized controlled trials of lidocaine infusion for perioperative pain.

Author	Condition treated	Study method	Number of subjects	Intervention	Outcome	Conclusion	Adverse events
Koppert	Major abdominal surgery	Prospective, randomized, and double-blinded study	40	IV lidocaine 2% (bolus 1.5 mg/kg in 10 min followed by an IV infusion of 1.5 mg/kg/h), vs. saline placebo. The infusion started 30 min before skin incision and was stopped 1 h after the end of surgery.	Postoperative pain ratings (numeric rating scale of 0–10), morphine consumption (patient-controlled analgesia).	Patients who received lidocaine reported less pain during movement and needed less morphine during the first 72 h after surgery -Opioid-sparing effect was most pronounced on the third postop day, IV lidocaine may have a true preventive analgesic activity, most likely by preventing the induction of central hyperalgesia.	No adverse events reported.
Wu	Laparoscopic cholecystectomy	Double-blind and randomized.	100	Co-treatment with dextromethorphan (DM) and intravenous lidocaine. (a) chlorpheniramine maleate (CPM) IM 20 mg and IV normal saline (N/S); (b) DM 40 mg IM and IV N/S (c) CPM 20 mg IM and IV lidocaine 3 mg/kg/h (d)DM40 mg IM and IV lidocaine All treatments were administered 30 min before skin incision.	Visual analog scale pain scores at rest and during coughing, time to meperidine request, total meperidine consumption, and the time to first passage of flatus after surgery.	DM group exhibited the best pain relief and fastest recovery of bowel function. Patients in the DM and lidocaine groups had significantly better pain relief than those in the CPM group.	No adverse events-related to the lidocaine infusion, except an occasional arrhythmia in 1 patient.
Kuo	Colonic surgery for colon cancer	Randomized	60	Thoracic epidural (TE) and IV lidocaine. TE group received lidocaine 2 mg kg followed by 3 mg.kg/h epidurally and an equal volume of IV normal saline. The lidocaine group received the same amount of lidocaine IV and normal saline epidurally. The control group received normal saline via both routes. All started 30 min before surgery and were continued throughout.	Cytokines IL6, IL8, and IL1RA. Return of bowel function. VAS pain scores at rest (A) and during coughing.	TE and IV lidocaine lower opioid consumption, allow earlier return of bowel function and lesser production of cytokines. TE was superior than IV lidocaine during 72 h after colonic surgery.	None reported, 3 patients had occasional bradycardia in the IV lidocaine group.
Kaba	Laparoscopic colectomy	Randomized placebo controlled	40	IV lidocaine (bolus injection of 1.5 mg/kg at induction of anesthesia, then a continuous infusion of 2 mg/kg/h intraoperative and 1.33 mg/kg /h for 24 h postoperatively Control: Saline.	Postoperative pain scores, opioid consumption, fatigue scores time to first flatus, defecation, and hospital discharge	Improvement in postop analgesia, fatigue, and bowel function. These benefits are associated with a significant reduction in hospital stay	Nausea 4 patient in saline 1 patient in lidocaine group, Vomiting 2 saline group, none lidocaine group
Herroeder	Abdominal surgery	Double-blinded, randomized, and placebo- controlled trial	60	IV lidocaine bolus (1.5 mg/kg) followed by a continuous lidocaine infusion (2 mg/min) until 4 h postoperatively.	Length of hospital stay, gastrointestinal motility, and the inflammatory response after colorectal surgery.	Lidocaine significantly accelerated return of bowel function and shortened length of hospital stay by one day. No difference could be observed in daily pain ratings	Wound healing and surgically related skin irritation in both lidocaine and placebo group
Martin	Total hip arthroplasty	Prospective two-center, randomized, double-blinded study	60	1.5 mg/kg IV bolus in 10 min then 1.5 mg /kg /h IV infusion or saline started 30 min before incision, stopped at 1h after skin closure.	Postoperative pain and modified nociceptive pain threshold	No significant difference between lidocaine and placebo on pain scores, pressure pain thresholds, area of hyperalgesia, and maximal degree of active hip flexion	No adverse events reported.
Lauwick	Laparoscopic cholecystectomy	Randomized and observer-blinded	50	At induction of anesthesia the control group (n=25) received fentanyl 3 μg.kg^−1^ while the lidocaine group received fentanyl 1.5 μg.kg^−1^ and a bolus of lidocaine 1.5 mg.kg^−1^ followed by a continuous infusion of lidocaine 2 mg.kg^−1^.hr^−1^.	The amount of fentanyl required in the PACU to establish and to maintain visual analogue scale pain scores <3.	Reduction in opioid consumption in the PACU and intraoperative requirements of desflurane.	No adverse events reported.
McKay	Ambulatory Surgery	Randomized double blind, placebo-controlled trial	67	At induction, all patients received 1.5 mg/kg of lidocaine by slow IV push. The lidocaine infusion (2 mg/kg/h or equivalent volume of saline as placebo), started immediately after induction of anesthesia and continued until 1 h after arrival in the PACU.	Pain and time to discharge from recovery	Length of postanesthesia care unit (PACU) stay did not differ between groups. Intraoperative opioid use was significantly less in the lidocaine group, both in the PACU and during the total study period but not after discharge.	No adverse events reported.
El-Tahan	Cesarean delivery	Randomized	90	Lidocaine 1.5 mg.kg^−1^ IV. bolus 30 min before induction, followed by an infusion of 1.5 mg.kg^−1^.h^−1^ until 1 h after surgery (n=45), or saline placebo (n=45).	Hemodynamic and hormonal responses.	Perioperative lidocaine is safe and effective in attenuating the maternal stress response to surgery for cesarean delivery.	No adverse events reported.
Yardine	Transabdominal hysterectomy	Randomized, placebo-controlled	60	Lidocaine + PCEA group received an IV bolus injection of 2 mg/kg lidocaine followed by a continuous IV infusion of 1.5 mg/kg/h saline. PCEA group a bolus and infusion of saline. Surgery ensued 20 min after lidocaine bolus. At completion of surgery, the lidocaine and saline infusions were terminated	Pain intensity, VAS scores at rest and during coughing in the first 8 hours. Immune reactivity during the postoperative period	Improves immediate postoperative pain management and reduces surgery-induced immune alterations.	No adverse events reported.
Baral	Upper abdominal surgery	Randomized	60	Lidocaine 2% (intravenous bolus 1.5 mg/kg followed by an infusion of 1.5 mg/kg/h), and 30 patients received normal saline. The infusion started 30 min before skin incision and stopped 1 h after the end of surgery.	Postoperative pain intensity at rest and movement, analgesic requirement diclofenac assessed at the interval 15 minutes for 1 h then 4 hourly up to 24 h.	Lidocaine decreases postoperative pain intensity, and reduces the postoperative analgesic consumption	No adverse events reported.
Cui	Thoracic surgery	Randomized	40	Lidocaine (33.0 mcg/kg/min and Saline control in propofol-remifentanil-based anesthesia.	Postoperative pain and morphine requirements. Pain scoring a four-point verbal rating scale, and a visual analogue scale. Morphine requirement in the PACU and morphine consumption via PCA.	Reduction in morphine requirements, postoperative pain and intraoperative propofol use.	No adverse events reported.
Bryson	Abdominal hysterectomy	Randomized, blinded placebo-controlled trial	90	IV bolus lidocaine of 1.5 mg/kg followed by an infusion of 3 mg/kg/hr, Control matching placebo.	The primary outcome discharge from hospital on or before the second postoperative day (POD2). Secondary outcomes: opioid use, pain scores, quality of recovery, and recovery of bowel function	Intraoperative administration of intravenous lidocaine did not reduce hospital stay or improve objective measures of analgesia and recovery.	Subjective symptoms of local anesthetic toxicity (lightheadedness, tinnitus, dysguesia, etc.) were reported by 21 (46%) of the control patients compared with only 11 (26%) of the lidocaine patients.
Kang	Inguinal herniorrhaphy	Prospective, randomized, double-blind, placebo-controlled	64	IV bolus 1.5 mg/kg lidocaine followed by a continuous lidocaine infusion of 2 mg/kg per hour.	Visual analogue scale pain scores, fentanyl consumption and the frequency at which analgesia	Total fentanyl consumption (patient-controlled plus investigator-controlled rescue administration) and the total number of button pushes were significantly lower in the lidocaine group than in the control group. It is concluded that intravenous lidocaine injection reduced post-operative pain after inguinal herniorrhaphy, is easy to administer and may have potential to become routine practice for this type of surgery.	The frequency of nausea was significantly lower in the lidocaine group than in the control group, but vomiting rates did not differ. Not reported any other complications.
Wongyingsinn	Elective Laparoscopic Colorectal surgery	Randomized Controlled Trial	60	Thoracic epidural analgesia (TEA group) or IV lidocaine infusion (IL group) (1 mg/kg per hour) with patient-controlled analgesia morphine for the first 48 hours after surgery.	The primary outcome was time to return of bowel function. Postoperative pain intensity, time out of bed, dietary intake, duration of hospital stay, and postoperative complications were also recorded.	Intraoperative and postoperative IV infusion of lidocaine in patients undergoing laparoscopic colorectal resection using an ERP had a similar impact on bowel function compared with thoracic epidural analgesia.	No adverse events related to lidocaine. Readmission rate of 23% in both groups.
Wasiak	Burn	Randomized double-blind, placebo-controlled, cross-over trial	45	Lidocaine of 1.5 mg/kg/body weight followed by two boluses of 0.5 mg/kg at 5-min intervals followed by a continuous infusion. During the control condition, 0.9% sodium chloride was administered at an equivalent volume	Primary end points included pain intensity as measured by verbal rating scale (VRS), time to rescue analgesia, opioid requests and consumption and overall anxiety and level of satisfaction.	The clinical benefit of intravenous lidocaine for pain relief during burn wound dressing changes in terms of overall pain scores and opioid consumption was unremarkable.	29% (13 pts) complained of nausea and vomiting. 1 pt reported twitchiness.
Grady	Laparoscopic Abdominal Gynecologic Procedures	Randomized double-blind, placebo controlled, cross-over trial	50	Lidocaine 1mg/kg bolus in both groups followed by an infusion of lidocaine 2 mg/kg/hr. vs. placebo saline which was stopped 15–30 minutes before skin closure.	1. VAS pain score on postoperative day 3 2. Morphine requirements in PACU 3. Return of bowel function.	Intraoperative lidocaine infusion improves postoperative pain levels and shortens time to return of bowel function	1 patient had protracted nausea and vomiting requiring readmission.
Wuethrich	Laparoscopic renal surgery	Randomized, double-blinded placebo-controlled	60	Lidocaine 1.5 mg kg bolus during induction of anesthesia, followed by an intraoperative infusion of 2 and 1.3 mg kg/h for 24 h postoperatively	Primary outcome was the length of hospital stay. Secondary outcomes were readiness for discharge, opioid consumption, sedation, incidence of postoperative nausea and vomiting (PONV), return of bowel function and inflammatory and stress responses	Perioperative lidocaine administration over 24 h did not influence the length of the hospital stay, readiness for discharge, opioid consumption, return of bowel function or inflammatory and stress responses	2 patients in lidocaine group had a surgical complication (need for pyelonephrostomy), and another wound infection. Postoperative delirium in one patient in the control group. No cardiac or pulmonary complications were observed
Grigoras	Breast surgery.	Randomized, double-blinded placebo-controlled	36	Lidocaine 1.5 mg/kg bolus followed by a continuous infusion of lidocaine 1.5 mg/kg/h or an equal volume of saline. The infusion was stopped 1 hour after the skin closure	Pain scores and analgesic consumption at 2, 4, 24 hours, and then daily for 1 week postoperatively. Three months later, patients were assessed for persistent postsurgical pain (PPSP) and secondary hyperalgesia.	perioperative lidocaine decreases the incidence and severity of PPSP after breast cancer surgery. Prevention of the induction of central hyperalgesia is a potential mechanism.	No adverse events reported.
De Oliveira	Ambulatory Laparoscopic Surgery	Randomized, double-blind, placebo-controlled	70	Lidocaine 1.5 mg/kg bolus followed by a 2 mg/kg/h infusion until the end of the surgical procedure, or an equal volume of saline	Primary outcome was the Quality of Recovery 40 questionnaire at 24 hours after surgery. A 10-point difference represents a clinically relevant improvement in quality of recovery based on a previously reported values on the mean and range of the Quality of Recovery-40 score	Lidocaine group had a significant better quality of recovery than the control group. There was an inverse relationship between opioid consumption and the quality of recovery	No adverse events reported.
Kim	Lumbar surgery	Randomized, placebo-controlled clinical trial	51	IV lidocaine infusion 1.5-mg/kg bolus followed by a 2-mg/kg/h infusion until the end of the surgical procedure versus normal saline infusion as a placebo.	The primary outcome was the visual analog scale (VAS) (0–100 mm) pain score at 4 hours after surgery. The secondary outcomes was the frequency of the button (FPB) of PCA being pushed and fentanyl consumption after surgery	The VAS scores and fentanyl consumption were significantly lower in the lidocaine group compared to placebo at 48 h after surgery (p<.05). Total fentanyl consumption, total FPB, length of hospital stay, and satisfaction scores were also significantly lower in lidocaine group compared with placebo.	No adverse events reported.
Tauzin-Fin	Laparoscopic nephrectomy	A two-phase observational study	47	I.V. lidocaine (1.5 mg/kg/h) was introduced, in the second phase, during surgery and for 24 h post-operatively.	Post-operative pain scores, opioid consumption and extent of hyperalgesia were measured. Time to first flatus and 6 min walking test (6MWT) were recorded	Intravenous (I.V.) lidocaine reduced post-operative morphine consumption and improved post-operative pain management and post-operative recovery after laparoscopic nephrectomy which contributed to better post-operative rehabilitation.	No major adverse events reported.
Farag	Complex spine surgery	Randomized controlled	116	IV lidocaine (2 mg/kg/hr.) or placebo during surgery and in the PACU.	Pain scores, verbal response scale. Quality of life at 1 and 3 months using the acute short-form (SF) 12 health survey.	IV lidocaine significantly improves postoperative pain after complex spine surgery.	No serious adverse events reported.
Tikuišis	Hand assisted laparoscopic colon surgery	Randomized controlled	64	IV lidocaine (dose unclear) vs. placebo	Visual analogue scale (VAS) scores at 2, 4, 8, 12, and 24 h after surgery.	Lidocaine superior to placebo in pain score, return of bowel function, and length of hospital stay	No significant adverse events.
Peng	Supratentorial craniotomy	Randomized controlled	94	IV lidocaine (1.5 mg/kg) bolus and infusion at a rate of 2 mg/kg/h until the end of surgery	Numeric rating scale (NRS) in PACU.	Lidocaine significantly decreases the proportion of patients with acute pain after supratentorial tumor surgery in the PACU.	No significant adverse events.
Zengin	Elective laparotomy	Randomized controlled	80	4 groups Group C, placebo capsules and normal saline infusion Group L, placebo capsules and lidocaine 1 mg/kg intravenous bolus dose followed by 2 mg/kg/h group P, 150 mg oral pregabalin and normal saline infusion perioperative; and group PL, 150mg oral pregabalin and lidocaine 2 mg/kg/h.	Visual analogue scale (VAS) scores, analgesic consumption, side effects, time to mobilization, time to first defecation, time to discharge and patients’ satisfaction	Oral pregabalin and perioperative intravenous lidocaine infusion decreased postoperative VAS scores. Oral pregabalin decreased morphine requirement. Intravenous lidocaine infusion hastened gastrointestinal motility and mobilization, and decreased the incidence of nausea	No significant adverse events.

**Table 3:** Summary of case reports and studies of lidocaine infusion for cancer pain.

Author	Condition treated	Study method	Number of subjects	Intervention	Outcome	Conclusion	Adverse events
Buchanan	Opioid refractory metastatic renal cell cancer	Case report	1 case	Lidocaine 1.8 mg/kg at a rate of 0.8 mg/kg/h.	Treatment of opioid refractory cancer pain	Lidocaine improved patients pain and end of life care	Infusion needed to be repeated after 3 weeks due return of pain.
Ferrini	Neuroectodermal tumor Breast cancer Rectal adenocarcinoma	Retrospective cases series	6 cases	Lidocaine ranging from 10–80 mg/h. 2 cases subcutaneous, 4 case intravenous	Treatment of opioid refractory cancer pain	Lidocaine improved patients pain and end of life care	Lightheadedness which improved after dose reduction.
Thomas	Refractory cancer pain	Retrospective chart review 0f 768 hospice patients	82 receiving intravenous lidocaine, 61 patients data was analyzed.	Lidocaine 1–2 mg/kg bolus followed by an infusion of 1mg/kg/h in 56 patients. 5 patients had no bolus	Treatment of opioid refractory cancer pain	50 patients had major improvement in pain out of which 44% had complete resolution of their pain 5 patients had partial response 6 patients had no benefit	No serious side effects reported, 26% had lethargy and somnolence.
Sharma	Refractory cancer pain	Randomize double blinded placebo controlled crossover	50	Lidocaine 2 mg/kg bolus over 20 min. followed by 2 mg/kg over 1 h.	Magnitude and duration of pain relief.	Significant improvement in pain relief of mean duration of 9.3 ± 2.58 days Significant reduction in analgesic requirements.	Self-limited side effects in the form of perioral numbness, tinnitus, sedation, lightheadedness and headache.
